# Statins attenuate PD-L1 sorting to small extracellular vesicles dependent on ubiquitin-like 3 modification

**DOI:** 10.1038/s41598-025-27789-x

**Published:** 2025-12-15

**Authors:** Hiroshi Ageta, Yoshihisa Shimada, Tadahiro Nagaoka, Kazuki Takenaka, Yusuke Yoshioka, Kohtaro Konno, Ryosuke Amemiya, Kumiko Nagase, Keisuke Hitachi, Takanori Onouchi, Masahiko Watanabe, Takahiro Ochiya, Kunihiro Tsuchida

**Affiliations:** 1 Division for Therapies Against Intractable Diseases, Center for Medical Science, Fujita Health University, Toyoake, 470-1192 Aichi Japan; 2 Department of Surgery, Tokyo Medical University Hospital, Shinjyuku-ku , Tokyo 160-0023 Japan; 3 Department of Molecular and Cellular Medicine, Institute of Medical Science, Tokyo Medical University, Shinjyuku-ku, Tokyo 160-0023 Japan; 4 Department of Anatomy, Hokkaido University Faculty of Medicine, Sapporo, 060-8638 Japan; 5 Open Facility Center, Research Promotion Headquarters, Fujita Health University, Toyoake, 470-1192 Aichi Japan; 6 Department of Medical Technology, Faculty of Medical Sciences, Shubun University, Ichinomiya, 491-0938 Aichi Japan

**Keywords:** UBL3, Statins, PD-L1, Small extracellular vesicles, Immunotherapy, Posttranslational modification, Ubiquitylation, Cancer immunotherapy, Cancer, Molecular medicine

## Abstract

Small extracellular vesicles (sEVs) mediate cell-to-cell communication by carrying RNAs and proteins. Ubiquitin-like 3 (UBL3) functions as a posttranslational modification factor, regulating protein sorting to sEVs. Programmed cell death ligand 1 (PD-L1) binds to programmed cell death 1 (PD-1) on immune cells, suppressing their function. Although immune checkpoint inhibitors, anti-PD-L1 and anti-PD-1 antibodies, have improved cancer treatment, efficacy remains limited (~ 25%). Per recent studies, PD-L1-containing sEVs are elevated in cancer patients, contributing to impaired immunotherapy responses. Herein, we discovered that PD-L1 is modified by UBL3 and that its sorting to sEVs is regulated by UBL3. Furthermore, we found that statins, commonly prescribed for hypercholesterolemia, inhibit UBL3 modification, thereby reducing PD-L1 sorting to sEVs. Among patients with a high tumor proportion score, serum levels of PD-L1-containing sEVs were significantly lower in those using statins. Consistently, bioinformatic analysis revealed that UBL3 and PD-L1 expression levels affect lung cancer survival. Integrating statins into existing combination therapies may therefore offer a promising strategy to enhance immunotherapy efficacy.

**Supplementary Information:**

The online version contains supplementary material available at 10.1038/s41598-025-27789-x.

## Introduction

The majority of proteins are subject to diverse post-translational modifications, including by ubiquitin, SUMO, and NEDD8^1–6^. Investigating novel post-translational regulatory mechanisms is crucial, as it can inform conceptually new strategies for drug development. Exosomes, a subtype of sEVs, are secreted by various cell types. Multivesicular bodies (MVBs) contain intraluminal vesicles, which are released as exosomes upon fusion with the plasma membrane. sEVs, including those derived from cancer cells, play a role in various diseases^7^. The ubiquitin-like 3 (UBL3), also known as membrane-anchored Ub-fold protein (MUB), was first identified in *Arabidopsis thaliana* and is a membrane localized protein by prenylation^8^. UBL3 has also been recognized as a biomarker for breast cancers^9^. Our recent findings indicate that, unlike ubiquitin, UBL3 modifies target proteins via disulfide bonds. Additionally, UBL3 regulates protein sorting to sEVs and interacts with molecules involved in tumor progression/metastasis molecules and neurodegenerative disease-related proteins^3,10–12^.

PD-L1 is a transmembrane protein expressed on the surface of various cell types, including cancer cells. PD-L1 binds to its receptor, PD-1, on the surface of T cells, leading to the inhibition of T-cell activation. Cancer immunotherapy is highly effective when immune checkpoint inhibitors, such as anti-PD-L1 and anti-PD-1 antibodies are used. However, its efficacy remains modest (~ 25% response rate), and the underlying mechanisms are not fully understood^13,14^. PD-L1 undergoes various post-translational modifications, including phosphorylation, ubiquitination, and glycosylation. These modifications alter its degradation rate, membrane trafficking, and conformational stability, thereby regulating its stability on the cell surface and binding affinity to PD-1. As a result, the T-cell inhibitory activity of PD-L1 may vary and directly influencing sensitivity to immune checkpoint inhibitors. Drug resistance may also be acquired through these modification^15^. For this reason, combination therapies with immune checkpoint inhibitors are being developed^16–22^. The extrafacial expression of PD-L1 on sEVs is reportedly elevated in patients with cancer, contributing to impaired cancer immunotherapy responses^23–26^. Furthermore, PD-L1-containing sEVs exert immune inhibitory effects and promote tissue repair^27^. Understanding the mechanism by which PD-L1 is sorted into sEVs is therefore a critical issue in improving cancer immunotherapy. However, their sorting mechanism to sEVs is still unknown. Our previous research has shown that approximately 60% of the proteins sorted to sEVs are influenced by UBL3^10^.

In the current study, we report that PD-L1 is modified by UBL3, and UBL3 enhances the PD-L1 sorting to sEVs. Intriguingly, we also find that UBL3 modification is inhibited by statins. Furthermore, we show a significant reduction in PD-L1 levels in the serum sEVs of statin users compared to non-users among lung cancer patients. Collectively, this study reveals a novel post-translational modification and sorting mechanism for PD-L1.

## Results

### PD-L1 is modified by UBL3

**Fig. 1:**
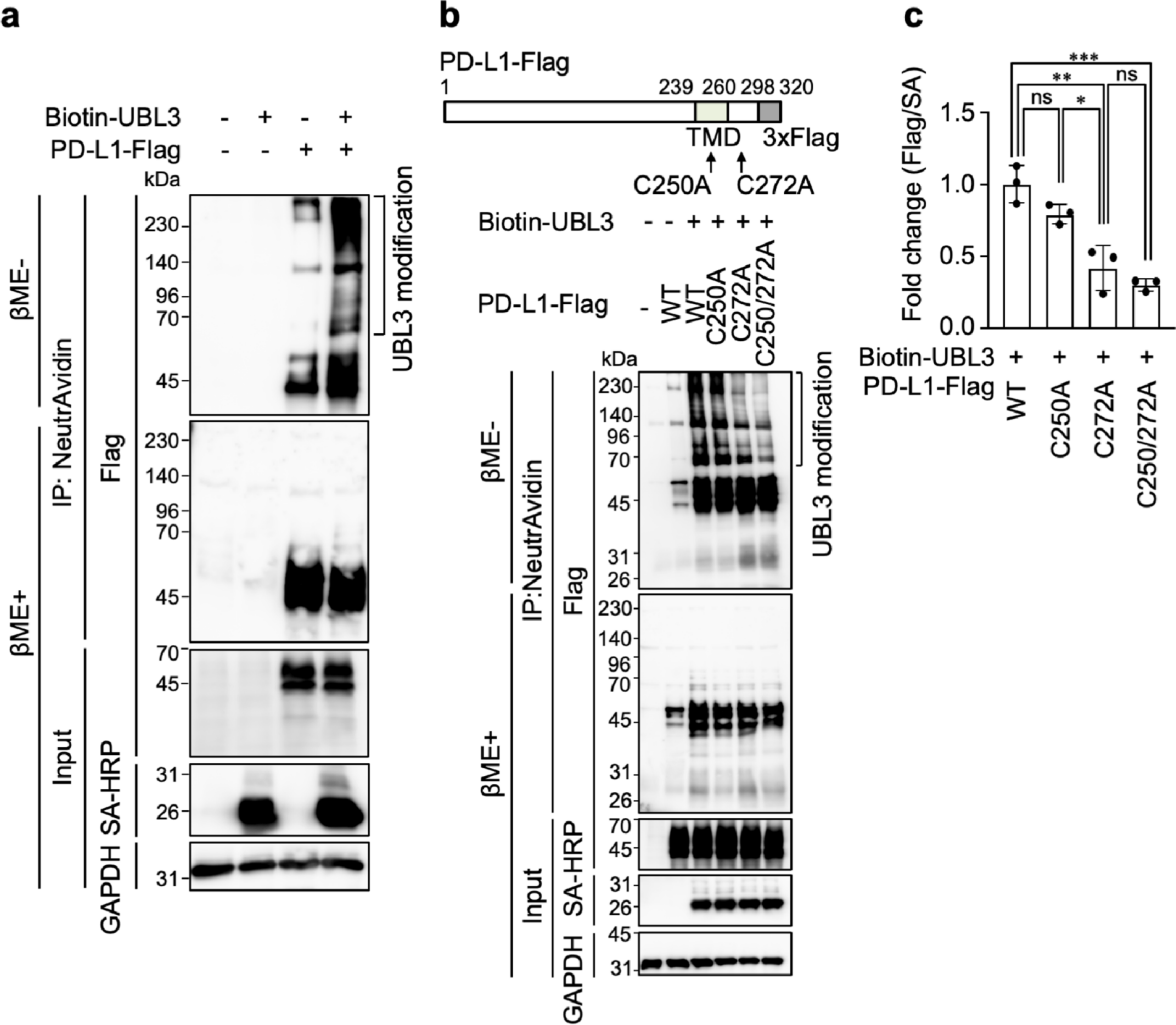
PD-L1 is modified by UBL3. (**a**) Co-immunoprecipitation (IP) analysis of using MDA-MB-231 cell lysates expressing Flag-tagged PD-L1 (PD-L1-Flag) and biotinylated-tagged UBL3 (Biotin-UBL3). (**b**) Co-IP was performed using cell lysates expressing PD-L1-Flag or PD-L1 mutants-Flag (C250A, C272A, C250/272A) along with Biotin-UBL3 in MDA-MB-231 cells. IB analysis was conducted using the indicated antibodies. (**c**) the relative intensity of UBL3 modification (Flag/SA-HRP) calculated as the ratio of UBL3-modified PD-L1 signals (> 70 kDa) to input SA-HRP signals, normalized to the mean of cells transfected with Biotin-UBL3 and wild-type PD-L1. Data are presented as mean ± s.e.m., with dots representing individual experiments. One-way ANOVA with Tukey’s multiple comparisons test. ns, not significant, *P < 0.05, **P < 0.001, ***P < 0.0005, PD-L1 vs. C250A, *P* = 0.1708; PD-L1 vs. C272A, *P* = 0.0009; PD-L1 vs. C250/272A, *P* = 0.0002; C250A vs. C272A, *P* = 0.0135; C272A vs. C250/272A, *P* = 0.5847; TMD, transmembrane domain; Flag, Flag-tag; βME-, without 2-mercaptoethanol; βME+, with 2-mercaptoethanol; SA-HRP, streptavidin-horseradish peroxidase.

We first examined whether PD-L1 is modified by UBL3. Unlike ubiquitin, UBL3 binds to its substrate via a disulfide bond; therefore, UBL3 modification was assessed using immunoprecipitation (IP). MDA-MB-231 breast cancer cells, which were previously used for the UBL3 modification assay^10^, were transfected with PD-L1-Flag and Biotin-UBL3, purified using streptavidin beads, and analyzed by immunoblotting with anti-Flag antibodies. The molecular weights of PD-L1-Flag and Biotin-UBL3 were approximately 45 and 26 kDa, respectively. In immunoprecipitates purified from cells co-transfected with PD-L1-Flag and Biotin-UBL3 under non-reducing conditions, signals were observed above 70 kDa. These signals disappeared when the immunoprecipitates were treated with 2-mercaptoethanol (βME+), indicating that PD-L1 is modified by UBL3 (Fig. 1a). PD-L1 contains two cysteine residues (C250 and C272) in its transmembrane and cytoplasmic domains, respectively; therefore, we constructed three PD-L1 mutants: C250A, C272A and C250/272A. UBL3 modification was minimally affected in the C250A mutant but reduced in the C272A mutant. Since the effect on the C250/272A double mutant tended to be reduced, but was comparable to that on the C272A mutant, C272 was considered the primary UBL3 modification site of PD-L1 (Fig. 1b). We quantified the UBL3-modified PD-L1 bands (Fig. 1c). The analysis confirmed that both C272A and C250/272A mutants exhibited significantly reduced UBL3 modification compared with WT, whereas no significant difference was observed between WT and C250A or between C272A and C250/272A. These results further support the conclusion that C272 is the major UBL3 modification site of PD-L1, with only a minor contribution from C250. In summary, PD-L1 is modified by UBL3 via the cysteine residues in its cytoplasmic domain.

### UBL3 regulates PD-L1 sorting to sEVs

**Fig. 2:**
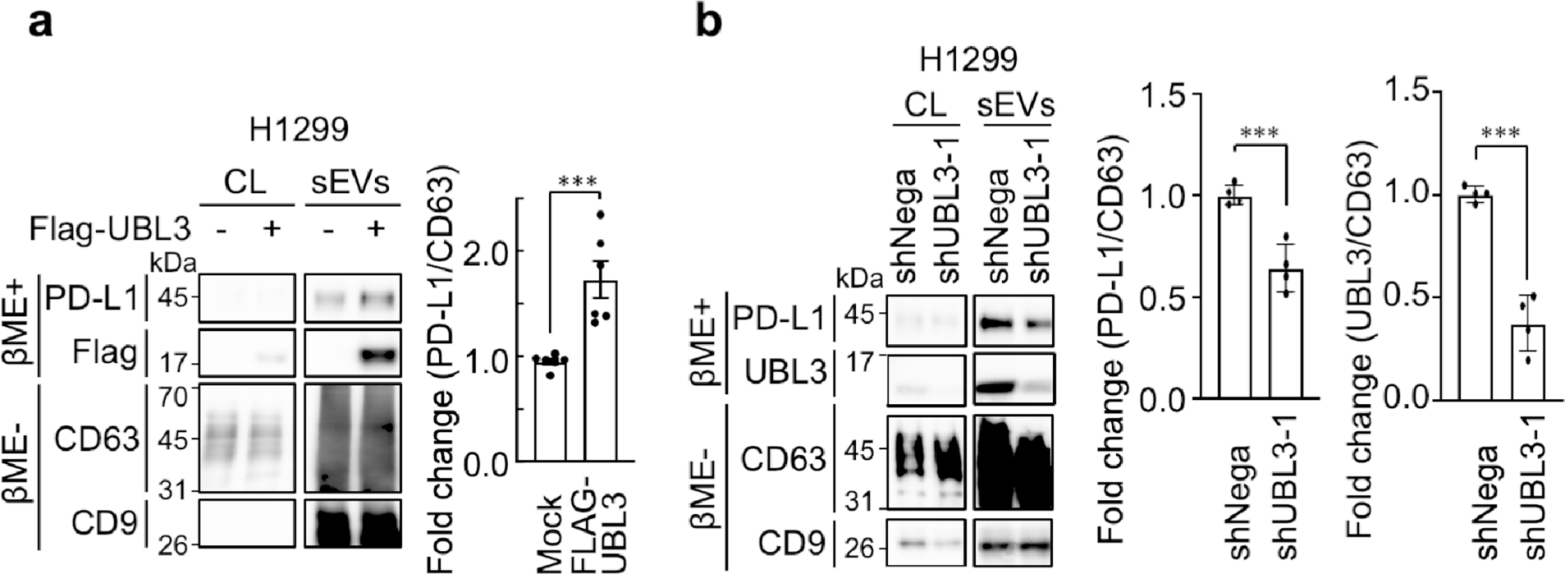
UBL3 regulates PD-L1 levels in sEVs. (**a**) Immunoblot (IB) analysis. The cell lysates and sEVs from the conditioned medium of H1299 cells transfected with 3xFlag-UBL3 vectors were blotted with various antibodies. (**b**) IB analysis. The cell lysates and sEVs from the stable UBL3 knockdown H1299 cell lines were blotted with various antibodies. CD9 and CD63 were used as sEV markers, as they are tetraspanin proteins abundantly expressed on the sEV surface^7^. Relative intensity of PD-L1 in sEVs was calculated as PD-L1/CD63 and presented as fold change, normalized to the mean value of the respective control (mock for a; shNega for b). Data are presented as mean ± s.e.m., with dots representing individual experiments. Two-tailed unpaired t-test. ***P < 0.005.

A previous study demonstrated that PD-L1 is sorted to sEVs released from WM9 melanoma cells^23^. We examined whether UBL3 is involved in this process and found that, while overexpression of UBL3 did not affect endogenous PD-L1 levels in cell lysates, it significantly increased PD-L1 levels in sEVs (Supplementary Fig. S1). We previously reported a significant increase in PD-L1 levels in the serum sEVs of patients with non-small cell lung cancer (NSCLC)^26^. Consistent with this, we observed elevated PD-L1 levels in the sEVs derived from H1299 human NSCLC cells upon UBL3 overexpression (Fig. 2a). In these experiments, CD9 and CD63, which are tetraspanin family proteins commonly enriched on the sEV surface, were used as standard markers to validate sEV purification^7^. To further validate the quality of purified sEVs, nanoparticle tracking analysis^28^ and electron microscopy revealed that the vesicles displayed a uniform diameter of approximately 150 nm (Supplementary Fig. S2). Moreover, we established stable UBL3 knockdown H1299 cell lines (Supplementary Fig. S3) and showed a significant reduction in PD-L1 levels in sEVs compared to controls (Fig. 2b). Together, these results indicate that UBL3 regulated the sorting of PD-L1 to sEVs in NSCLC cells.

### Inhibition of UBL3 modification and PD-L1 sorting by Statins

**Fig. 3:**
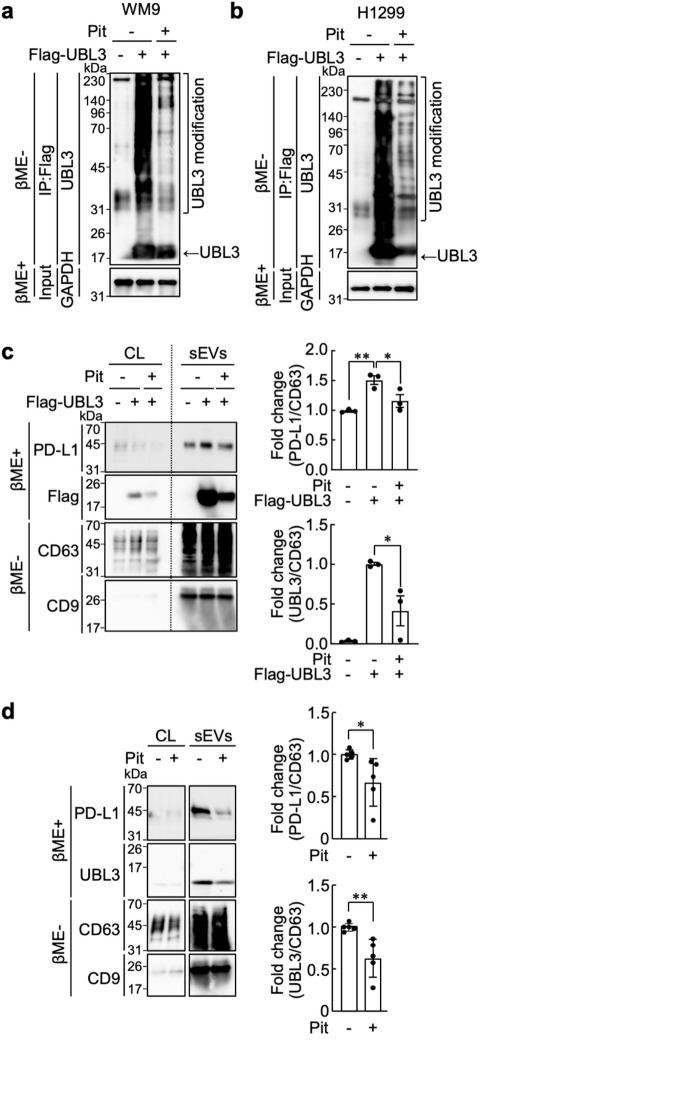
Inhibition of UBL3 modification and PD-L1 sorting by statins. (**a** and **b**), Effect of pitavastatin treatment on UBL3 modification in WM9 (**a**) and H1299 (**b**) cells. Five hours after transfection, 10 µM pitavastatin was added. (**c** and **d**), IB analysis of the cell lysates and sEVs from the conditioned medium of H1299 cells transfected with 3xFlag-UBL3 vectors (**c**) or untransfected (**d**) were blotted with various antibodies. Five hours after gene transfection, 0.2 µM pitavastatin was added. Pit, Pitavastatin. Right panels, the relative intensity of PD-L1 in sEVs (c: PD-L1/CD63 [upper] normalized to the mean of mock − untreated control, and UBL3/CD63 [lower] normalized to the mean of Flag-UBL3 − untreated control; d: PD-L1/CD63 [upper] and UBL3/CD63 [lower], both normalized to the mean of untreated control). Data are presented as mean ± s.e.m., with dots representing individual experiments. As inhibition of UBL3 modification was observed even at low concentrations of pitavastatin (Supplementary Fig. S4b), a low concentration of pitavastatin was used in sEV purification experiments. IB analysis using the indicated antibodies. Flag, Flag-tag; GAPDH, internal control; CD63 and CD9, sEVs markers; PD-L1, endogenous PD-L1; UBL3, endogenous UBL3. βME−, without 2-mercaptoethanol. βME+, with 2-mercaptoethanol. (**c**), one-way ANOVA with Tukey’s multiple comparisons test. (**d**), Two-tailed unpaired t-test. *P < 0.05, **P < 0.01.

In our previous study, two cysteine residues (C113 and C114) in the C-terminal region of UBL3 were found to be important for the UBL3 modification activity^10^. UBL3 is prenylated at C114, allowing it to anchor to the membrane^8^. The localization of UBL3 to MVBs depends on UBL3 modification^10,12^. Therefore, we investigated the effects of statins, which inhibit prenylation and are widely used as therapeutic agents for hyperlipidemia, on UBL3 modification. UBL3 modification was strongly inhibited by treatment with mevastatin in MDA-MB-231 cells (Supplementary Fig. S4a). Furthermore, all six clinically used statins (pitavastatin, rosuvastatin, atorvastatin, lovastatin, simvastatin, and fluvastatin) inhibited UBL3 modification (Supplementary Fig. S4b). Similar effects were observed both in WM9 melanoma and H1299 NSCLC cells (Fig. 3a, b). Additionally, in MDA-MB-231 cells co-transfected with PD-L1-Flag and Biotin-UBL3, statin treatment reduced UBL3 modification of PD-L1 (Supplementary Fig. S5). We then administered pitavastatin to UBL3-overexpressing H1299 cells and examined its effect on the sorting of endogenous PD-L1 to sEVs. PD-L1 sorting to sEVs, which was enhanced by UBL3, was significantly inhibited by pitavastatin treatment. Furthermore, we found that the sorting of UBL3 to sEVs was significantly reduced by pitavastatin administration (Fig. 3c). When statin was administered to H1299 cells that did not overexpress UBL3, we observed a significant reduction in the levels of endogenous UBL3 and PD-L1 contained in sEVs (Fig. 3d).

### Statin use is associated with lower sEV PD-L1 levels in lung cancer patients with high tumor PD-L1 expression

**Fig. 4:**
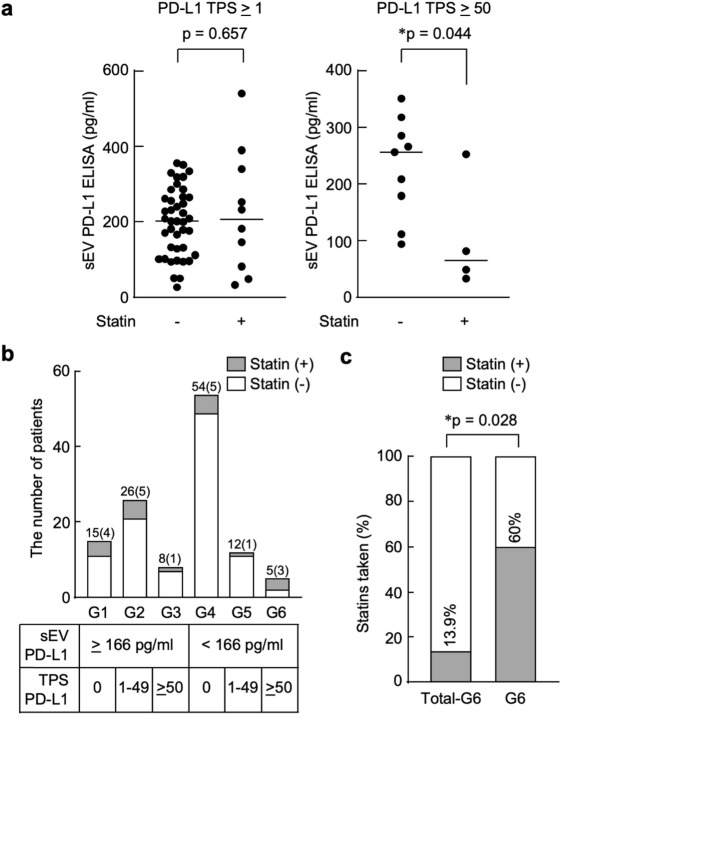
Statin use is associated with lower sEV PD-L1 levels in lung cancer patients with high tumor PD-L1 expression. (**a**) ELISA analysis of PD-L1-containing sEVs. Left, patients with tumor PD-L1-positivity (TPS *≥* 1%; statin (-), *n* = 41; statin (+), *n* = 10). Right, patients with high tumor PD-L1 positivity (TPS *≥* 50%; statin (-), *n* = 9; statin (+), *n* = 4). Two-tailed unpaired t-test. The horizontal line indicates the median value for each group. (**b**) Classifications of patients into six groups based on tumor PD-L1 levels and PD-L1-containing sEV concentrations. Groups 1–6 were defined according to tumor PD-L1 expression categories (TPS 0%, 1–49%, or *≥* 50%) combined with sEV PD-L1 concentrations above or below the cutoff value of 166 pg/mL. The numbers in parentheses indicate the number of patients taking statins. (**c**) Proportion of patients taking statins was highest in Group 6 (G6). white bar, statin (-). grey bar, statin (+). Fisher’s exact test. The number of patients taking statins is as follows: each drug: atorvastatin (4), simvastatin (1), pitavastatin (3), pravastatin (6), and rosuvastatin (5). In G6: pitavastatin (1), pravastatin (1), and rosuvastatin (1).

We previously reported that PD-L1 levels were significantly elevated in the serum sEVs of patients with NSCLC^26^. Therefore, we conducted a retrospective study to investigate the effect of statins on PD-L1 levels in serum sEVs from patients with lung cancer. The correlation between PD-L1-containing sEV levels and statin intake in patients with tumor PD-L1 positivity is shown in Fig. 4a. Tumor PD-L1 expression was determined by immunostaining, and sEV PD-L1 levels were measured by ELISA. Although no significant correlation was observed between sEV PD-L1 levels and statin intake in the entire population of tumor PD-L1-positive (tumor proportion score [TPS] *≥* 1%) patients (*p* = 0.657), a significant inverse correlation between sEV PD-L1 and statin intake was observed in patients with high tumor PD-L1 levels (TPS *≥* 50%). Among patients with high tumor PD-L1 expression, statin users had lower sEV PD-L1 levels (Fig. 4a, right panel). In our previous study, we identified an optimal cut-off value of 166 pg/mL for PD-L1-containing sEVs, which was relevant to tumor PD-L1 positivity. Using this threshold, we assessed the relationship between PD-L1-containing sEVs, tumoral PD-L1 expression, and statin intake. The 120 patients were then classified into six groups based on the tumor PD-L1 expression and levels of PD-L1-containing sEVs (Fig. 4b). Specifically, Groups 1–6 represent patient subsets stratified by two parameters: (i) tumor PD-L1 expression (TPS 0%, 1–49%, or *≥* 50%) and (ii) serum sEV PD-L1 concentrations above or below the 166 pg/mL cutoff identified in our previous study. The proportion of patients taking statins was highest in Group 6 (G6), which included patients with sEV PD-L1 levels below 166 pg/mL and high tumor PD-L1 expression (TPS *≥* 50%). A significant difference in statin intake was observed between the G6 and the rest of the patient groups (Fig. 4c).

### UBL3 and PD-L1 expression levels influence survival in lung cancer patients

**Fig. 5:**
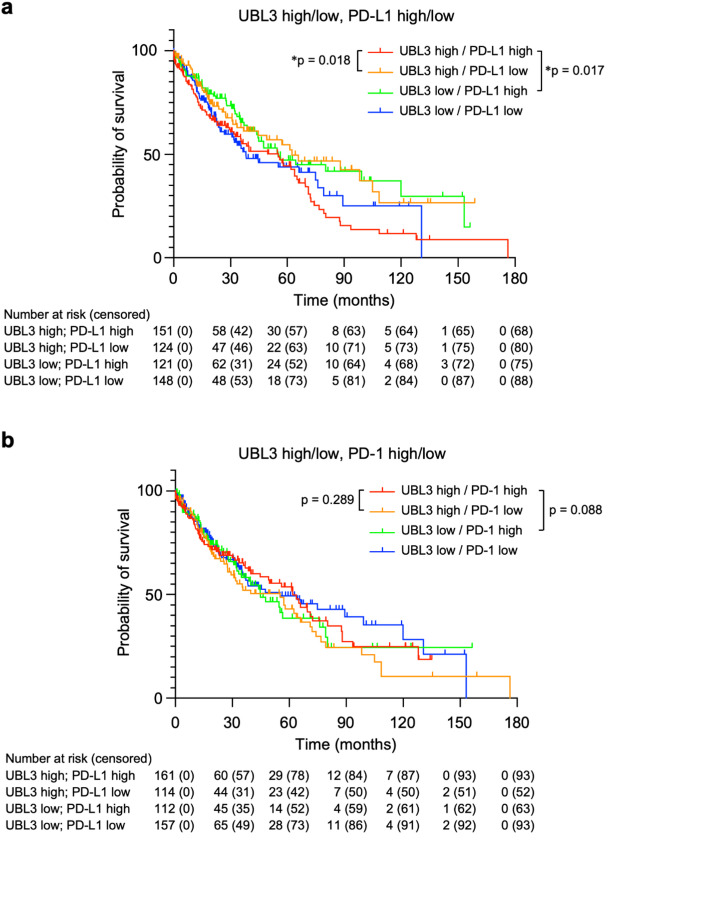
UBL3 and PD-L1 expression levels influence survival in lung cancer patients. (**a**) Kaplan–Meier survival curves for the lung squamous cell carcinoma cohort, stratified by UBL3 and PD-L1 expression. (**b**) Survival curves for the same cohort, stratified by UBL3 and PD-1 expression. Hazard ratios and p-values from log-rank (Mantel–Cox) tests are summarized in Supplementary Figure S7a.

Our experimental results demonstrated that UBL3 modification plays a critical role in controlling PD-L1 sorting to sEVs and that this pathway can be pharmacologically targeted by statins. These findings raised the question of whether the interplay between UBL3 and PD-L1 also influences disease progression and patient prognosis. To evaluate the prognostic significance of UBL3 and PD-L1 expression levels, we analyzed the survival among lung squamous cell carcinoma patients in a public database. Our analysis revealed that the individual expression levels of UBL3 or PD-L1 did not significantly affect the survival rate (Supplementary Fig. S6a, b and S7a). However, when the expression levels of UBL3 and PD-L1 were combined, a significant difference in survival was observed. Specifically, patients in the high UBL3 expression and low PD-L1 expression (UBL3 high/PD-L1 low) and low UBL3 expression and high PD-L1 expression (UBL3 low/PD-L1 high) groups had significantly higher survival rates than those in the high UBL3 expression and high PD-L1 expression (UBL3 high/PD-L1 high) group (Fig. 5a and Supplementary Fig. S7a). These findings suggest that the combined expression levels of UBL3 and PD-L1, rather than their individual expression levels, play a critical role in determining survival outcomes in lung cancer. In contrast, a similar analysis of PD-1 did not reveal a significant association between its expression level and survival rate, either alone or in combination with UBL3 (Fig. 5b and Supplementary Fig. S6c and S7a).

## Discussion

PD-L1 is regulated by various post-translational modifications, including phosphorylation and glycosylation and acetylation^29–34^. In addition, its ubiquitination is regulated by E3 ubiquitin ligases and deubiquitinating enzymes, which in turn affect PD-L1 stability and subsequently influence the suppression of T cell responses^35–39^. UBL3 is structurally similar to ubiquitin^8^, and interacts with E2 ubiquitin-conjugating enzymes and E3 ubiquitin ligases at the cell membrane^40,41^. In the present study, we found that PD-L1 is directly modified by UBL3 via the cysteine residue in its cytoplasmic domain, and that overexpression of UBL3 enhances its sorting to sEVs. We also found that statins, which are widely used in clinical practice, strongly inhibit UBL3 modification, and that treatment with pitavastatin attenuates PD-L1 sorting to sEVs.

In melanoma treatment, the pre-treatment level of PD-L1-containing sEVs were significantly higher in patients who did not respond to cancer immunotherapy^23^. In mouse experiments, PD-L1–containing sEVs have been shown to suppress T cell activity, and sEV-deficient tumor cells induce anti-tumor immunity^24^. Subsequent validation by other groups also reported high levels of PD-L1 in sEVs from melanoma patients^25^. Similarly, elevated levels of PD-L1 in sEVs have been reported in patients with NSCLC^26^. These findings suggest that an increase in PD-L1-containing sEVs impairs the therapeutic effects of cancer immune checkpoint inhibitors. In this current study, we elucidated the mechanism by which PD-L1 is sorted to sEVs via UBL3 modification and discovered that statins inhibit UBL3 modification. Numerous studies have investigated the use of statins as anticancer drugs^42^, and their anticancer effects are enhanced when combined with other anticancer drugs, such as carboplatin (a platinum-based drug) and erlotinib (a tyrosine kinase inhibitor)^43,44^. Studies in NSCLC have shown that combining statins with anti-PD-1/PD-L1 therapy improves therapeutic efficacy compared with anti-PD-1/PD-L1 therapy alone^45,46^. These findings align with the results of this study. Therefore, enhancing the efficacy of anti-PD-1/PD-L1 therapies is of significant public health importance. Various combination strategies have been explored, including chemotherapy, tyrosine kinase inhibitors, anti-TIGIT antibodies, IL-27 receptor agonists, and anti-GDF-15 antibodies, in conjunction with anti-PD-1/PD-L1 therapy^16–19,21,22^. In addition to statins, other clinically approved drugs have also been explored as combination partners for immune checkpoint inhibitors. Interestingly, metformin, a widely used antidiabetic drug, has also been shown to induce the phosphorylation and degradation of PD-L1^30^. Metformin has been reported to enhance the efficacy of immunotherapy when used in combination with immune checkpoint inhibitors by modulating the tumor microenvironment and angiogenesis^30,47,48^.

In the present study, we observed that pitavastatin suppressed PD-L1 sorting to sEVs at a concentration as low as 0.2 µM. This low dosage was intentionally chosen to approximate pharmacologically relevant exposure levels. Previous reports indicate that statins exert cytotoxic effects in lung cancer cells only at relatively high concentrations (5–100 µM) in vitro^49,50^, whereas in vivo administration (10–50 mg/kg) results in plasma concentrations of only a few micromolar levels^49,51^. Our findings therefore suggest that inhibition of UBL3-dependent PD-L1 sorting to sEVs can occur at sub-micromolar concentrations, within the range potentially achievable by therapeutic dosing in patients, and independent of overt cytotoxicity. This highlights a novel mechanism by which statins may exert antitumor or immunomodulatory effects at clinically relevant doses.

This study has several limitations. First, our experiments relied primarily on cell-based models, and we did not perform in vivo validation. Although the current results clearly demonstrate that UBL3 modification regulates PD-L1 sorting to sEVs and that this process can be inhibited by statins in vitro, it remains to be determined whether these effects translate into enhanced anti-tumor immune responses in vivo. Future studies employing appropriate animal models will be necessary to address this important question and to establish the therapeutic potential of statins as modulators of PD-L1 sorting to sEV. Second, the number of statin users in our retrospective cohort was relatively small (*n* = 4–10), which may limit the robustness of the conclusions. Although the proportion of patients receiving statin treatment in our cohort (15.8%, 68 ± 9 years; Fig. 4B) was comparable to the reported proportion of Japanese adults aged ≥ 60 years receiving dyslipidemia treatment (16.5%; dyslipidemia including hypercholesterolemia, which represents the primary indication for statin therapy)^52^, the absolute number of statin-treated patients remained limited. Third, our analysis did not differentiate between different types or dosages of statins, which could affect their biological impact on UBL3-dependent PD-L1 sorting. Future studies with larger cohorts and prospective designs will be required to address these issues and to further validate our findings.

In conclusion, we showed that UBL3 modification enhances PD-L1 sorting to sEVs, and statins inhibit this process, correlating with reduced PD-L1-containing sEVs. Taken together, our findings suggest that adding statins as UBL3 inhibitors to established dual regimens—such as anti-PD-1/PD-L1 therapy combined with chemotherapy or tyrosine kinase inhibitors—may offer a promising triple combination approach to further enhance therapeutic efficacy.

## Methods

### Cell cultures and reagents

Human non-small cell lung cancer H1299 cell (CRL-5803, ATCC), human melanoma WM9 cells (WM9-01-0001, Rockland) and human breast cancer cell MDA-MB-231(MDA-MB-231-luc-D3H2LN, Xenogen) were cultured in Roswell Park Memorial Institute (RPMI) 1640 medium (11875-093, Gibco) with 10% heat-inactivated fetal bovine serum (FBS; SH30910.03, Cytiva). HEK293T (RIKEN cell Banks) was cultured in Dulbecco’s modified Eagle’s medium (11965-092, Gibco) with 10% heat-inactivated FBS (SH30910.03, Cytiva). All cultures were incubated in 5% CO_2_ at 37 °C. All the cell lines were routinely tested for Mycoplasma contamination (EZ-PCR Mycoplasma Test kit, Biological Industries). The following statins were used for UBL3 modification experiments: Pitavastatin (S1759, Selleckchem), atorvastatin calcium (S2077, Selleckchem), rosuvastatin calcium (S2169, Selleckchem), simvastatin (S1792, Selleckchem), lovastatin (S2061, Selleckchem), fluvastatin sodium (S1909, Selleckchem), and mevastatin (M2537, Sigma).

### Plasmid construction

Biotin-UBL3 and 3xFlag-UBL (wild-type, C113A, C114A and C113/114A mutants) were generated as described previously^10^. The human PD-L1 (NP_054862) expression vector was subcloned into the pcDNA3 vector (Invitrogen) using standard molecular biology techniques and PCR^10^.

In the shRNA experiments, shNega (Target sequence: shNega, ATCCGCGCGATAGTACGTA) was used as a negative control. The plasmid pSUPER-mRFP-shNega was kindly provided by Natsumi Ageta-Ishihara^53^. pSUPER-mRFP1-entry was digested with BglII and HindIII, and shRNAs (Target sequence: shUBL3-1, AGGTCAGCAGTCCAAATAT; shUBL3-2, GGTCAGCAGTCCAAATATT) were inserted. The shNega, shUBL3-1, and shUBL3-2 fragments containing the H1 promoter were then excised and subcloned into a Sleeping Beauty plasmid pSBbi-GP lacking the EF1α promoter, resulting in the constructs shNega < pSB-GP2, shUBL3-1 < pSB-GP2, and shUBL3-2 < pSB-GP2. pSUPER-mRFP1-entry was a gift from Natsumi Ageta-Ishihara^53^. pSBbi-GP was a gift from Eric Kowarz (Addgene plasmid #60511)^54^. pCMV(CAT)T7-SB100 was a gift from Zsuzsanna Izsvák (Addgene plasmid #34879)^55^. The Sleeping Beauty system was described previously^54^.

### Antibodies

To expression of glutathione S-transferase (GST) fusion proteins, we subcloned cDNA fragments encoding mouse UBL3 (NM_001359199) into the pGEX-4T-1 plasmid (GE Healthcare). Immunization and affinity purification were performed as described previously^56^. Briefly, purified GST fusion protein (10 mg) was denatured using sodium dodecyl sulphate (SDS) sample buffer (31.2 mM Tris–HCl [pH 6.8], 2% SDS, 10% glycerol, 0.001% bromophenol blue, 2.5% 2-mercaptothanol), and subjected to electrophoresis on a 15% SDS-PAGE gel. The protein band was excised and homogenized in 10 mL of phosphate-buffered saline (PBS) using a Polytron homogenizer. The prepared antigen was then injected subcutaneously into rabbits. The antibodies used in the study were as follows: UBL3 (1:100; lab-generated; 1:1000; 14100-1-AP [Lot#00005139], Proteintech), Flag (1:1000; F3165, Sigma), streptavidin-HRP (1:5000; 19534-050, Invitrogen), GAPDH (1:1000; 2118, Cell Signaling), CD63 (1:1000; Ts63, Thermo), CD9 (1:1000; Ts9, Thermo), PD-L1 (1:500; 405.9A11, Cell Signaling), GFP (1:1000; 598, MBL) and horseradish peroxidase-conjugated IgGs (1:1000; 7076 and 7074, HRP-linked Antibody, Cell Signaling; 1:1000; 18–8816-31 and 18–8817-33, TrueBlot, Rockland).

### Transfection, immunoprecipitation and IB

H1299 cells were transfected with plasmids using Lipofectamine 3000 (L3000015, Thermo), while MDA-MB-231 cells and WM9 cells were transfected with plasmids using Lipofectamine 2000 (11668030, Thermo). The UBL3 modification assay was performed as previously described^10^. Briefly, IP was conducted 24 h post transfection. Cell lysis was performed without the addition of a commercial protease inhibitor cocktail or the reducing agent dithiothreitol. Cell lysates were incubated with 30 µL of Pierce High Capacity NeutrAvidin Agarose beads (29202, Thermo) for 18 h at 4 °C with rotation. After washing the beads, bound proteins were eluted in 40 µL of 2× sample buffer (100 mM Tris–HCl [pH 6.8], 4% SDS, 20% glycerol, and 0.01% bromophenol blue, without 2-mercaptoethanol) for 5 min at 95 °C (βME−). Subsequently, 18 µL of each sample was treated with 2 µL of 2-mercaptoethanol and incubated for 5 min at 95 °C (βME+). IB analysis was performed as described previously^57^. To detect UBL3 modification, TrueBlot was used to suppress signals derived from antibodies present in the immunoprecipitated samples. Chemiluminescence signals were detected using ECL Plus (32132, Thermo) and captured using LAS-4000 mini imaging system (FUJIFILM).

### The establishment of stable UBL3 knockdown H1299 cell lines

H1299 cells were transfected with shNega < pSB-GP2, shUBL3-1 < pSB-GP2, and shUBL3-2 < pSB-GP2 together with pCMV(CAT)T7-SB100 using Lipofectamine 3000. The next day, the medium was replaced. Cells were then selected with puromycin (2 µg/ml) containing medium. After 10 days of puromycin treatment, stable H1299 cell lines expressing shNega, shUBL3-1, and shUBL3-2 were established.

### Isolation of sEVs

PD-L1-containing sEVs were isolated from conditioned medium following the protocol described by Chen et al., with slight modifications^23^. In brief, conditioned medium was prepared using sEV-depleted FBS, obtained by centrifugation at 100,000 × g for 16 h. Following a 24-hour incubation, culture medium (approximately 55 mL from H1299 cells (12 × 10⁶), WM9 cells (12 × 10⁶), and MDA-MB-231 cells (13 × 10⁶)) was collected and subjected to centrifugation at 2,000 × g for 20 min at 4 °C. The resulting supernatant was further centrifuged at 16,500 × g for 45 min at 4 °C. The sEV pellet was harvested by ultracentrifugation at 100,000 × g for 120 min at 4 °C using a Beckman SW32Ti rotor. The pellet was resuspended in 1 mL PBS, washed, and re-collected via ultracentrifugation at 100,000 × g for 120 min at 4 °C using a Beckman TLA-110 rotor. A final wash with 1 mL PBS was followed by ultracentrifugation at 100,000 × g for 60 min at 4 °C before resuspension in PBS. Protein concentrations in the cell lysates were measured using the BCA assay kit (23227, Thermo). To quantify protein concentrations in sEV pellets, samples were diluted in 2% SDS and measured using the Micro-BCA assay kit (23235, Thermo).

### Gene expression and survival data analysis

Fragments per kilobase of transcript per million mapped reads (FPKM) and overall survival datasets from the Genomic Data Commons (GDC) and The Cancer Genome Atlas (TCGA) were retrieved and analyzed for Lung Squamous Cell Carcinoma (LUSC) cohort (*n* = 552) using UCSC Xena (https://xena.ucsc.edu/)^58^. The median FPKM value of each gene was used as the threshold to classify high- and low- expression groups (Supplementary Fig. S7b). Overall survival (OS) was measured from the day of treatment to the day of death from any cause or the day on which the patient was last known to be alive.

### Association between the level of PD-L1-containing sEV levels, tumor PD-L1 expression, and statin use

We previously assessed whether the level of PD-L1-containing sEVs in the serum could help predict the response to anti-PD-1 therapy in 120 patients with NSCLC who underwent pulmonary resection between January 2015 and December 2016 at Tokyo Medical University Hospital. This study was approved by the Institutional Review Board of Tokyo Medical University (SH4064), and informed consent for the use and analysis of clinical data was obtained preoperatively from each patient. Using data from these 120 patients, we evaluated the association between the level of PD-L1-containing sEV levels, tumor PD-L1 expression, and statin use. Statin use was determined by reviewing medical records.

The Human CD274/PD-L1 ELISA kit (ARG81929, Arigo) was used to quantify the level of PD-L1-containing sEVs concentration following the manufacturer’s protocol. Briefly, 96-well plates were incubated with standards at various concentrations alongside serum samples. After covering with antibodies, HRP-conjugated streptavidin was added and protected from light. The enzymatic reactions were developed, and the absorbance was measured at 450 nm using a microplate reader.

Immunohistochemical (IHC) staining for PD-L1 (E1L3N, #113684; CST) was performed on whole-section samples. PD-L1 staining was assessed using the Tumor Proportion Score (TPS). TPS was defined as the number of positive tumor cells divided by the total number of viable tumor cells multiplied by 100%.

### Nanoparticle tracking analysis

Nanoparticle concentration and size distribution were measured using the NanoSight LM10 instrument (Malvern Instruments, UK)^28^. Each sample was diluted 200- or 300-fold with 0.1 μm-filtered phosphate-buffered saline (PBS) to achieve the optimal particle concentration for analysis. For each measurement, a 60-second video was recorded under fixed settings (camera level: 13; detection threshold: 7).

### Electron microscopy for sEV observation

Ultramicroscopic analysis was carried out using a standard procedure with minor modifications^59^. In brief, purified sEVs were mixed in equal volumes with 4% paraformaldehyde in PBS and placed onto electron microscopy (EM) grids (Excel Support Film, Nisshin EM). After rinsing the grids with PBS, the sEVs were post-fixed for 5 min with 1% glutaraldehyde, followed by eight washes with ultrapure water at 2 min intervals. The grids were then negatively stained with 10% EM stain (Nisshin EM) and analysed using a transmission electron microscope (JEM-1400Flash, JEOL).

### Statistical analysis and data presentation

Statistical analyses were carried out using Prism 9.4.1 (GraphPad Software). T-test or one-way ANOVA with Tukey’s multiple comparisons test were respectively applied for comparisons between 2 groups and 3 or more groups. We used more than two independent samples for each experiment. Statistical methods, P values, sample sizes, and error bars were stated in the figure legends. OS curves were plotted using the Kaplan–Meier method, and differences in variables were determined using the log-rank test. The GraphPad Prism 10 (GraphPad Software) was used for survival analysis. Representative entire images of immunoblots are shown in Supplementary Figure S8, with boxed areas indicating cropped regions used in the designated figures.

## Supplementary Information

Below is the link to the electronic supplementary material.

Supplementary Material 1

## Data Availability

All datasets, resources and reagents are available upon request from the corresponding authors.
