# Supplementary material for: Statins attenuate PD-L1 sorting to small extracellular vesicles dependent on ubiquitin-like 3 modification
Source: Sci Rep. 2025 Dec 15;15:43802. doi: 10.1038/s41598-025-27789-x (PMC12706078; doi:10.1038/s41598-025-27789-x)
Supplement: Supplementary file 1 — Supplementary Material 1 [file 41598_2025_27789_MOESM1_ESM.docx]

**Supplementary Information**

**Statins attenuate PD-L1 sorting to small extracellular vesicles dependent on ubiquitin-like 3 modification**

Hiroshi Ageta^1*^, Yoshihisa Shimada^2^, Tadahiro Nagaoka^1^, Kazuki Takenaka^1^, Yusuke Yoshioka^3^, Kohtaro Konno^4^, Ryosuke Amemiya^2^, Kumiko Nagase^2^, Keisuke Hitachi^1^, Takanori Onouchi^5, 6^, Masahiko Watanabe^4^, Takahiro Ochiya^3^, Kunihiro Tsuchida^1*^

1. Division for Therapies Against Intractable Diseases, Center for Medical Science, Fujita Health University, Toyoake, Aichi 470-1192, Japan

2. Department of Surgery, Tokyo Medical University Hospital, Shinjyuku-ku, Tokyo 160-0023, Japan

3. Department of Molecular and Cellular Medicine, Institute of Medical Science, Tokyo Medical University, Shinjyuku-ku, Tokyo 160-0023, Japan

4. Department of Anatomy, Hokkaido University Faculty of Medicine, Sapporo 060-8638, Japan

5. Open Facility Center, Research Promotion Headquarters, Fujita Health University, Toyoake, Aichi 470-1192, Japan

6. Department of Medical Technology, Faculty of Medical Sciences, Shubun University, Ichinomiya, Aichi, 491-0938, Japan.


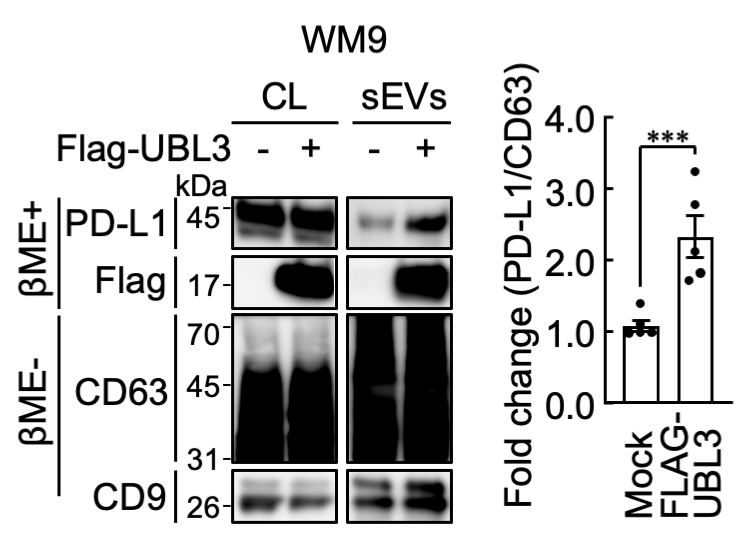


**Supplementary Figure S1 UBL3 overexpression increases PD-L1 levels in sEVs in WM9 cells.**

Immunoblot (IB) analysis of the cell lysates and sEVs from the conditioned medium of WM9 cells transfected with 3xFlag-UBL3 vectors were blotted with various antibodies. In the right panel, the fold change of PD-L1 expression in sEVs was calculated as PD-L1/CD63 values normalized to the mean of mock control. Data are presented as mean ± s.e.m., with dots representing individual experiments. Two-tailed unpaired t-test. ***P < 0.005.


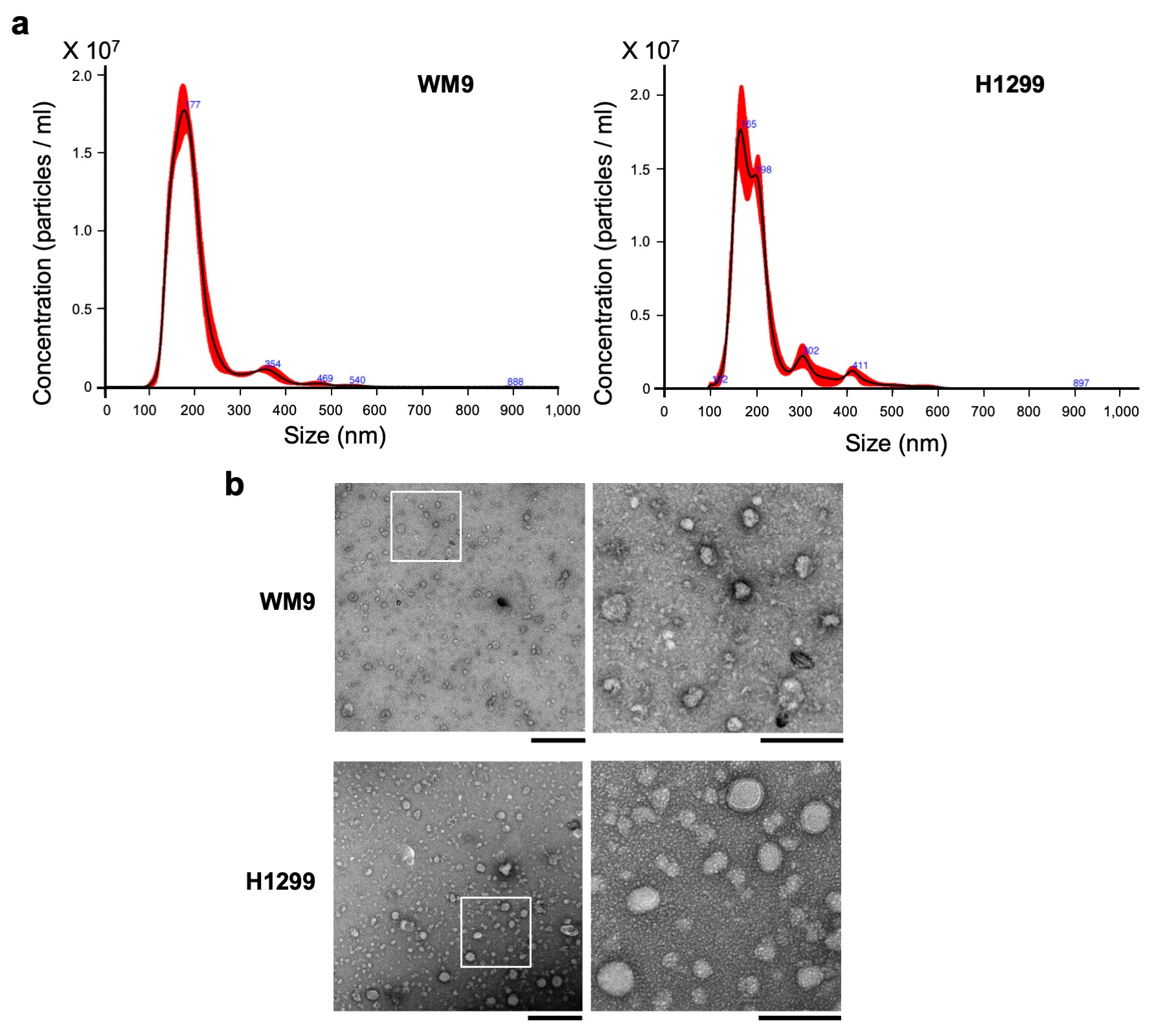


**Supplementary Figure S2 Validation of sEV characterization by nanoparticle tracking analysis and electron microscopy.**

(a) Nanoparticle tracking analysis (NTA) showing the size distribution of EVs derived from WM9 and H1299 cells. The vertical axis represents the EV particle concentration (×10⁷/mL), and the horizontal axis indicates particle size (nm). Data are presented as the mean (black line) ± standard deviation (SD, red line) of three independent samples.

(b) Electron microscopic analysis of the sEVs. Right panel, magnified regions from the white box in the left panel. Scale bars, 500 nm (left panels) and 200 nm (right panels).


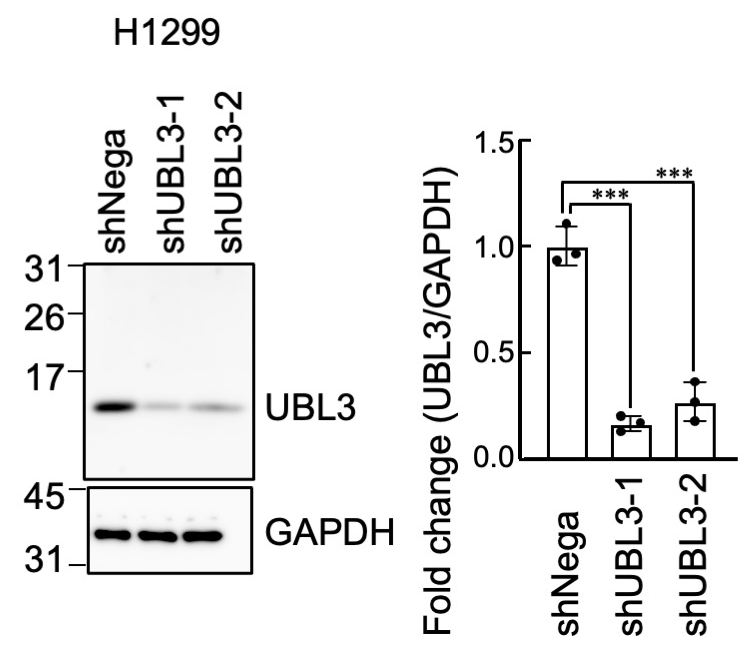


**Supplementary Figure S3 The establishment of stable UBL3 knockdown H1299 cell lines.**

IB analysis of the cell lysates from the stable UBL3 knockdown H1299 cell lines were blotted with various antibodies. UBL3, endogenous UBL3. GAPDH, internal control. In the right panel, the fold change of endogenous UBL3 in cell lysates was normalized to GAPDH and to the mean of shNega stable cells. Data are presented as mean ± s.e.m., with dots representing individual experiments. Tukey's multiple comparisons test. ***P < 0.0001.


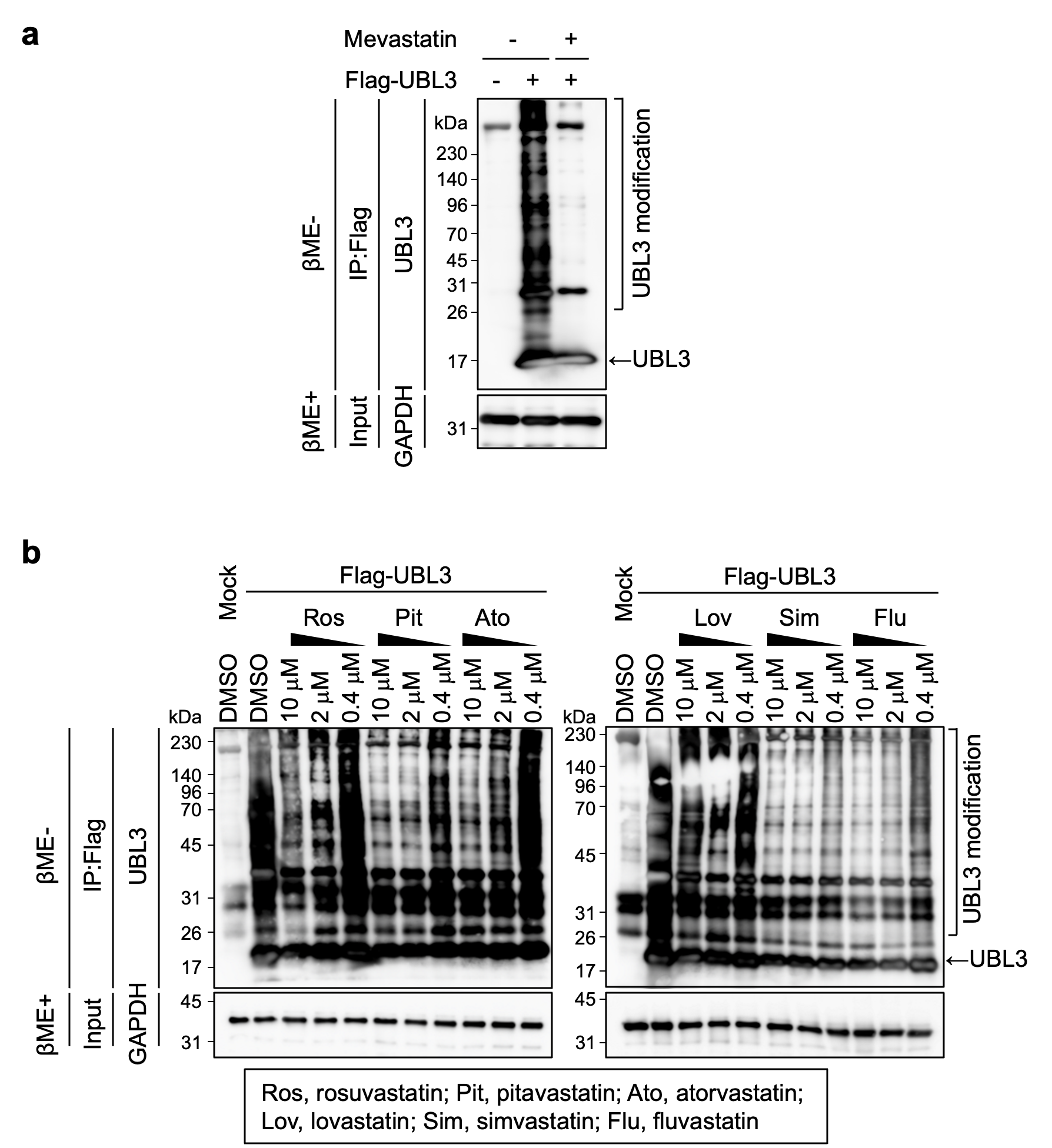


**Supplementary Figure S4 UBL3 modification is inhibited by statin treatment.**

(a) Effect of mevastatin treatment on UBL3 modification in MDA-MB-231 cells. UBL3 modification was detected by IP with anti-Flag antibodies in MDA-MB-231 cells transfected with 3xFlag-UBL3, followed by IB analysis with anti-UBL3 antibodies. Mevastatin (10 μM) was added 5 h after transfection. (b) Effects of different statins on UBL3 modification. UBL3 modification was detected by IP with anti-Flag antibodies in HEK293T cells transfected with 3xFlag-UBL3, followed by IB analysis with anti-UBL3 antibodies. After gene transfection, various statins with different concentrations were administered.


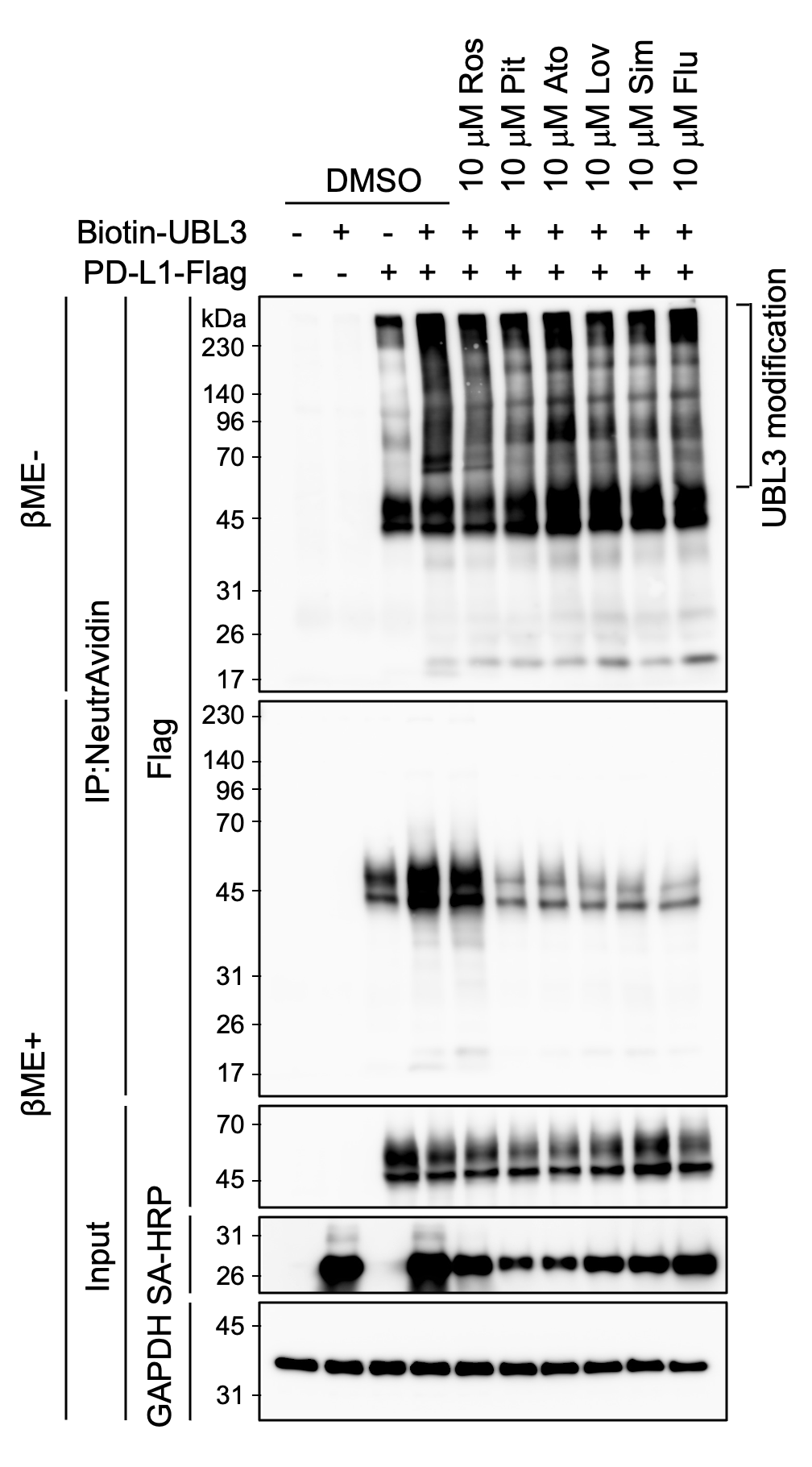


**Supplementary Figure S5**

Effect of various statin treatment on UBL3 modification of PD-L1. UBL3-modified PD-L1-Flag was purified using streptavidin beads from MDA-MB-231 cells transfected with Biotin-UBL3 and PD-L1-Flag and then detected by IB analysis with anti-Flag antibodies. Statins (10 μM) were added 5 h after transfection.


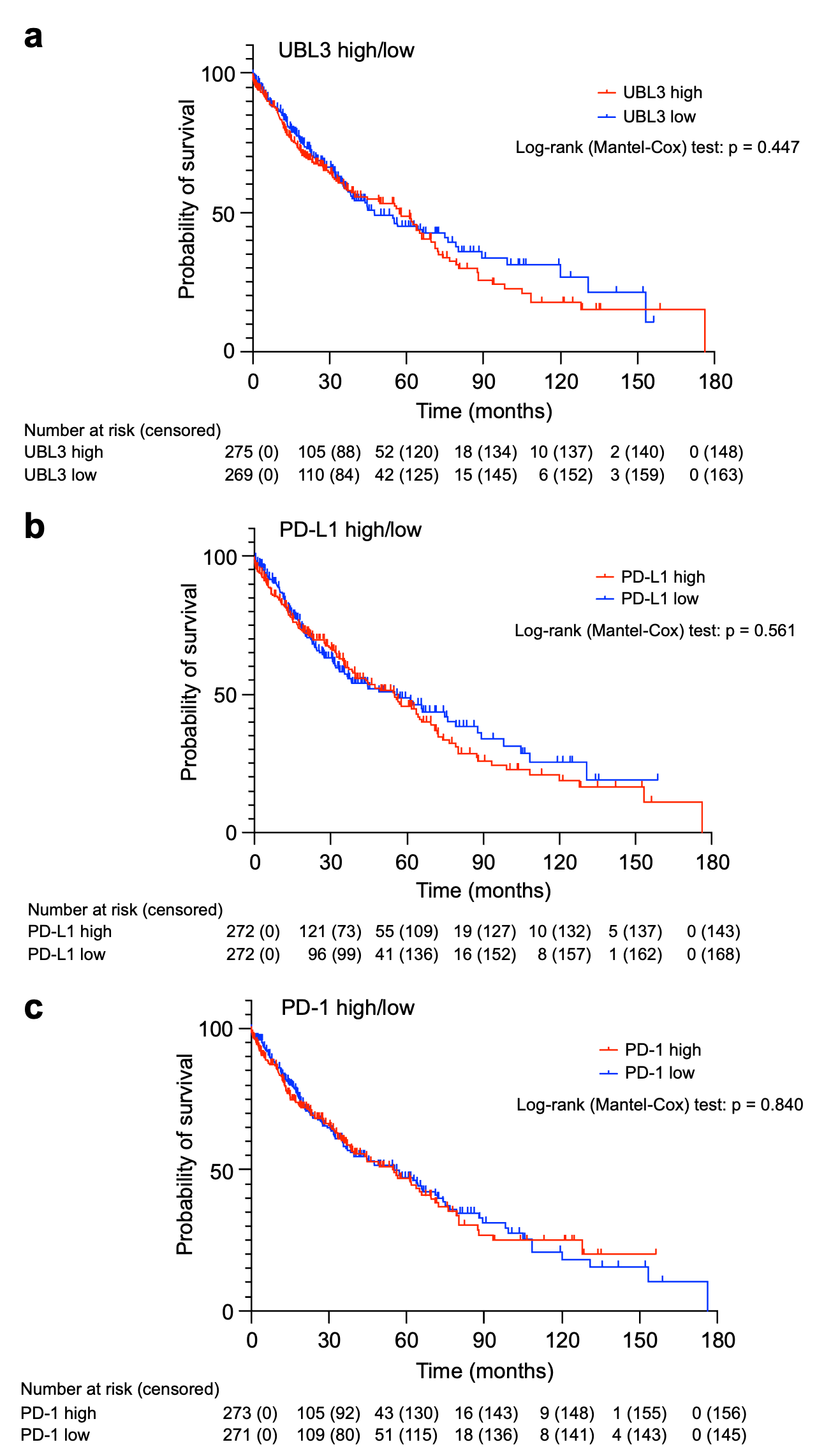


**Supplementary Figure S6 Kaplan–Meier survival plots of the Lung Squamous Cell Carcinoma cohort based on differences in single-gene expression differences.**

(a) Comparison between UBL3 high (red, log2(FPKM-upper quartile [UQ]+1) ≥ 3.446, n = 275) and UBL3 low (blue, log2(FPKM-UQ+1) < 3.446, n = 269). (b) Comparison between PD-L1 high (red, log2(FPKM-UQ+1) ≥ 2.069, n = 272) and PD-L1 low (blue, log2(FPKM-UQ+1) < 2.069, n = 272). (c) Comparison between PD-1 high (red, log2(FPKM-UQ+1) ≥ 1.152, n = 273) and PD-1 low (blue, log2(FPKM-UQ+1) < 1.152, n = 271). Hazard ratios and p-value from the log-rank (Mantel-Cox) test are also summarized in the Supplementary Figure S7a.


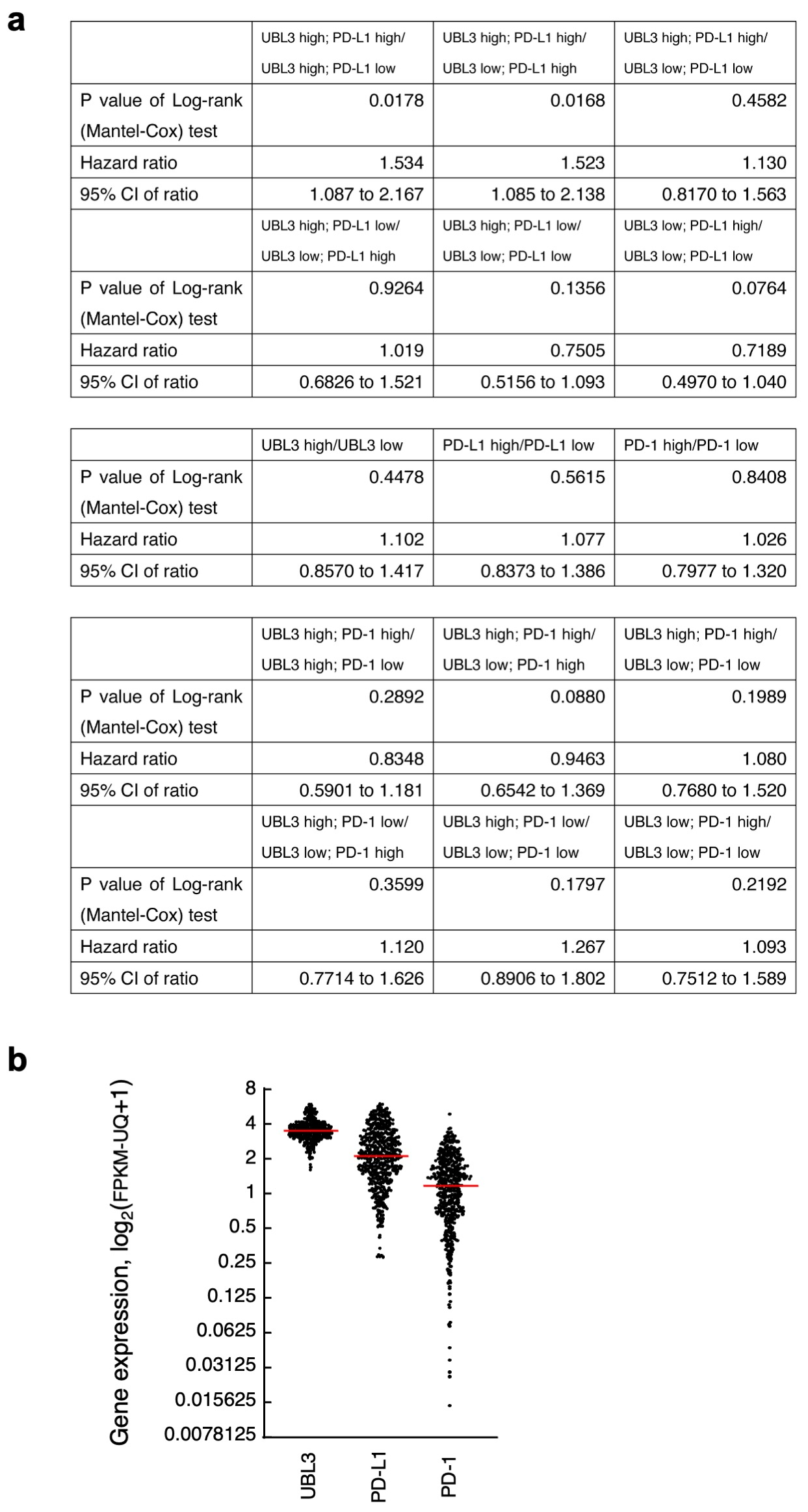


**Supplementary Figure S7 Gene expression RNA-seq analysis of the Lung Squamous Cell Carcinoma cohort (n = 552).**

(a) P-values of Log-rank test and hazard ratios for survival in GDC TCGA LUSC cohort based on differences in gene expression levels. (b) Scattered dot plots show UBL3, PD-L1, and PD-1 gene expression. The horizontal red line indicates the median expression of each gene (UBL3 = 3.446, PD-L1 = 2.069, PD-1 = 1.152).


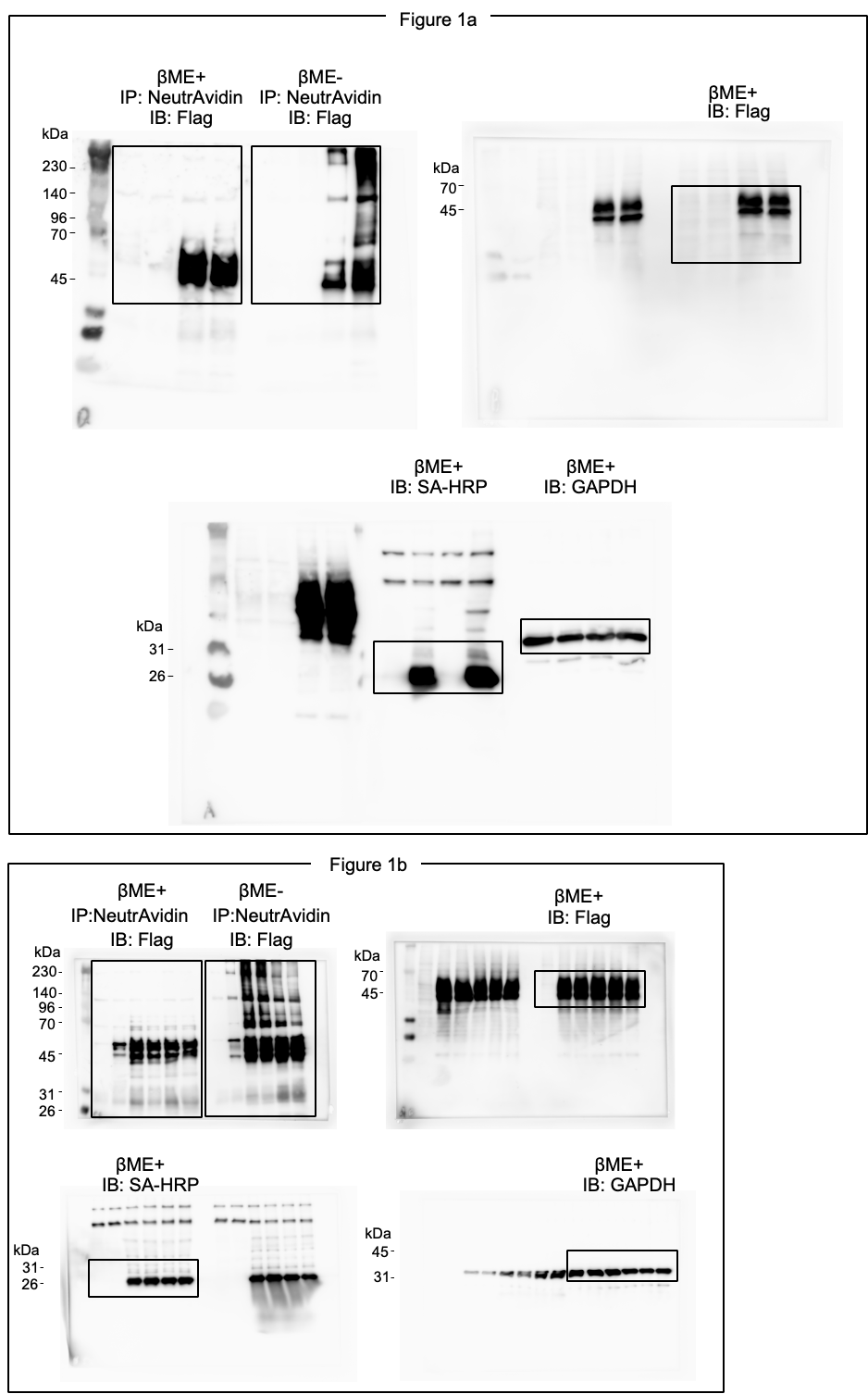


**
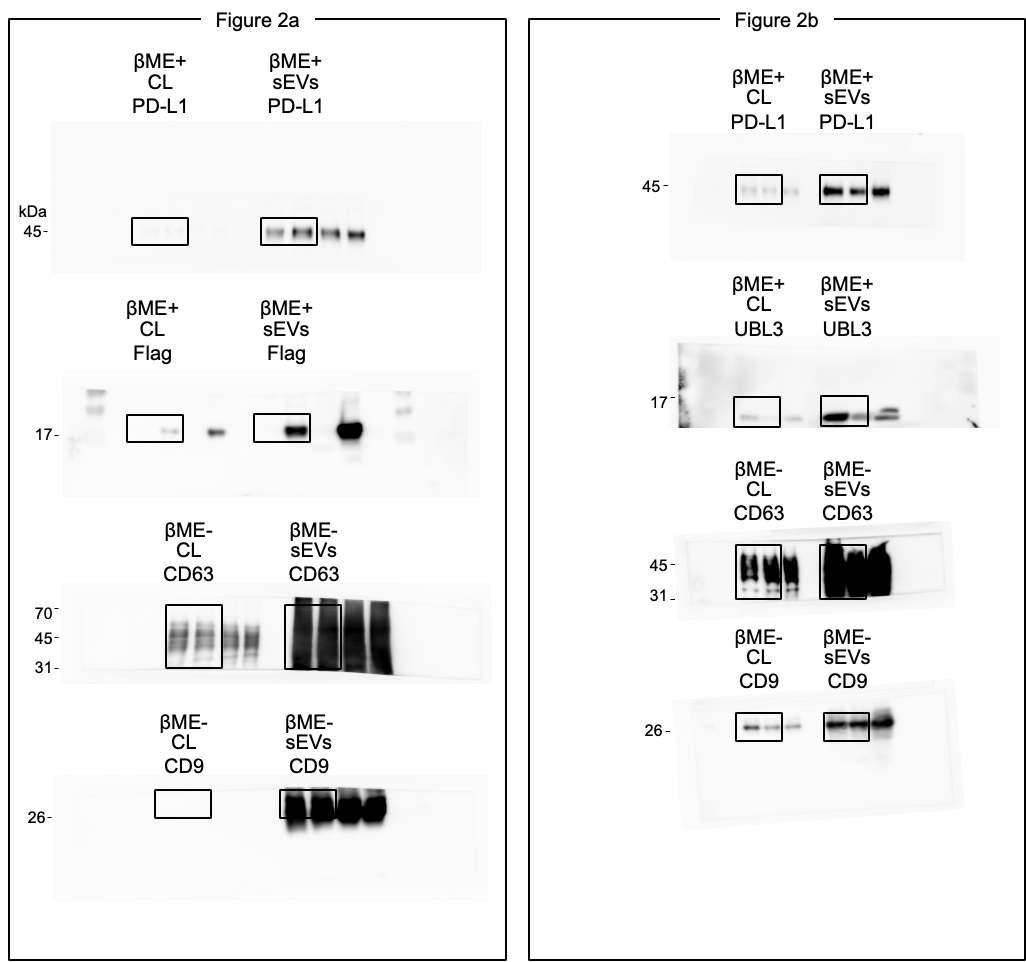
**

**
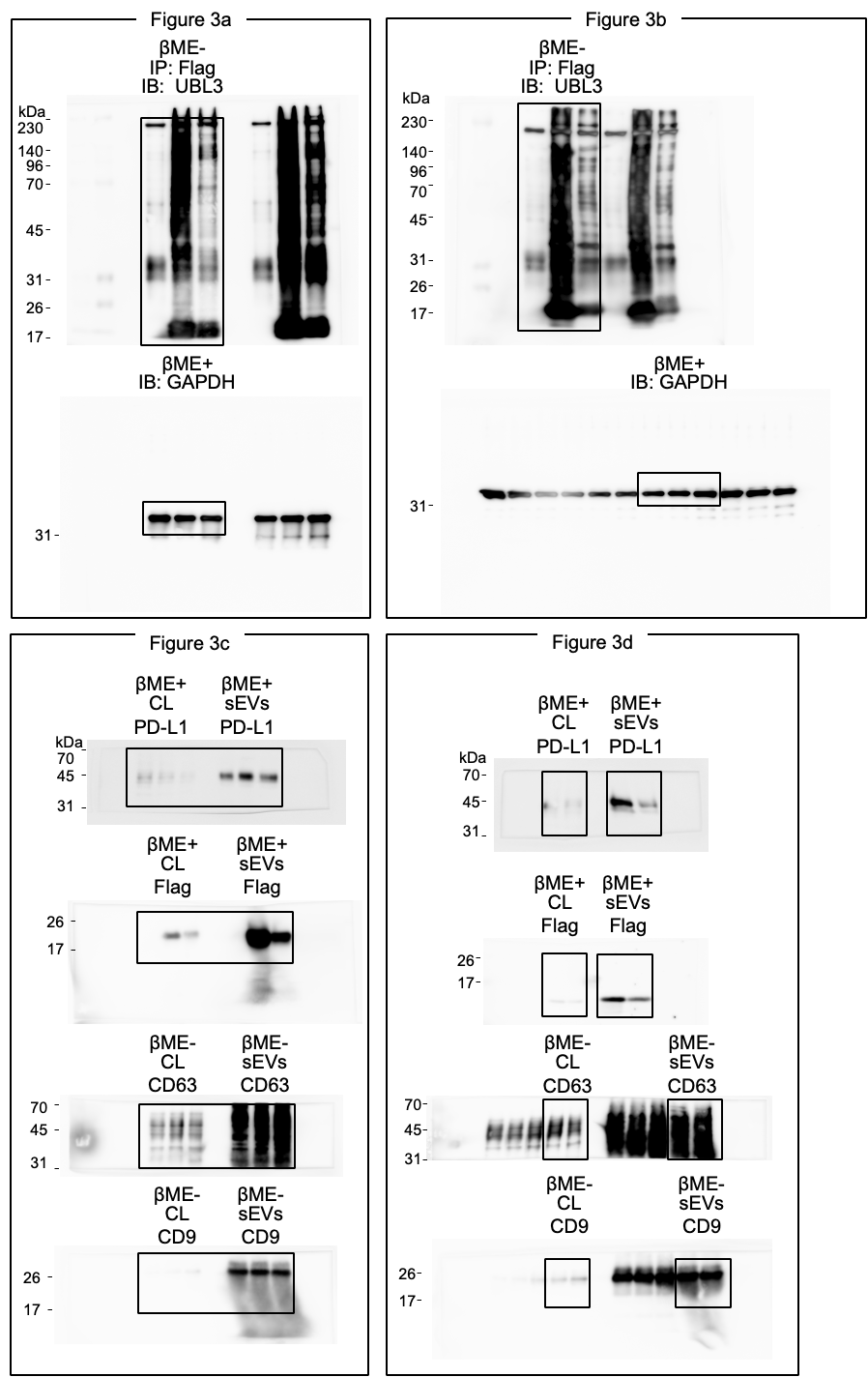
**

**
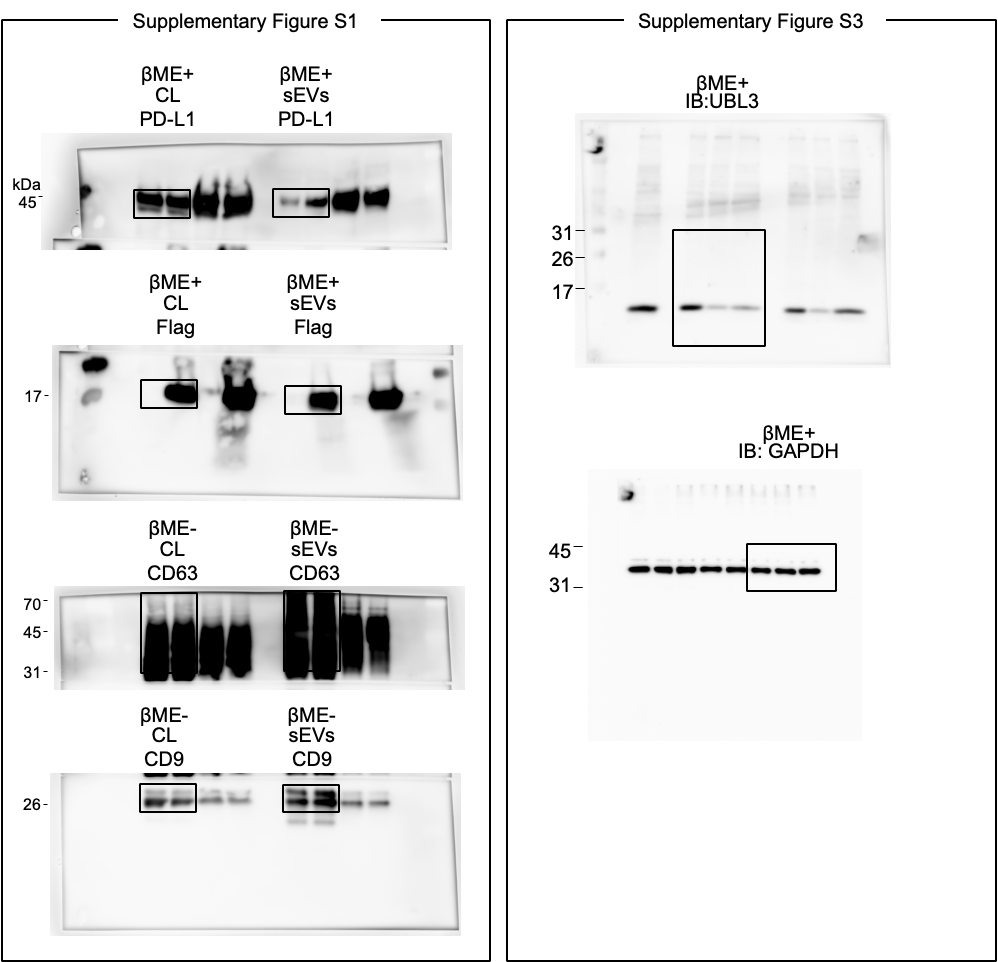
**

**
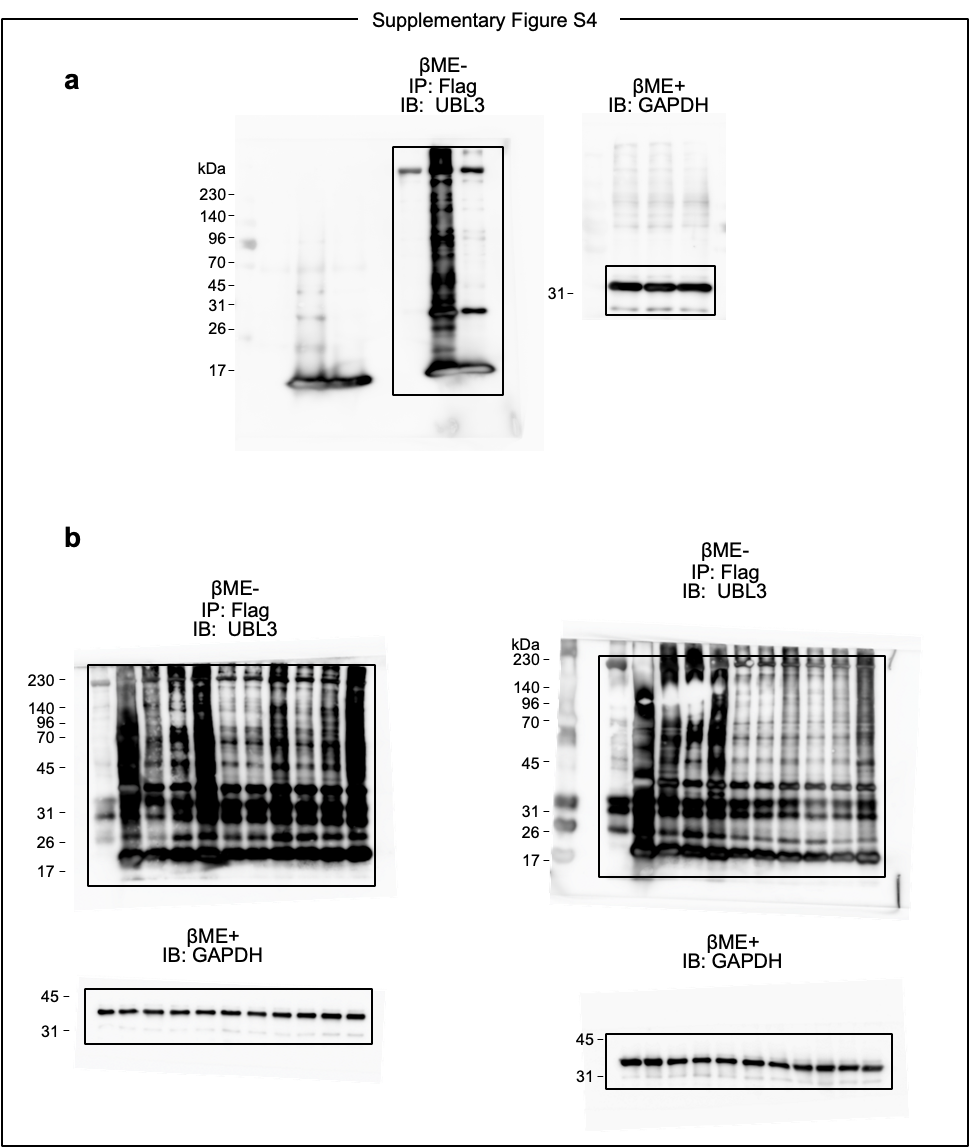
**

**
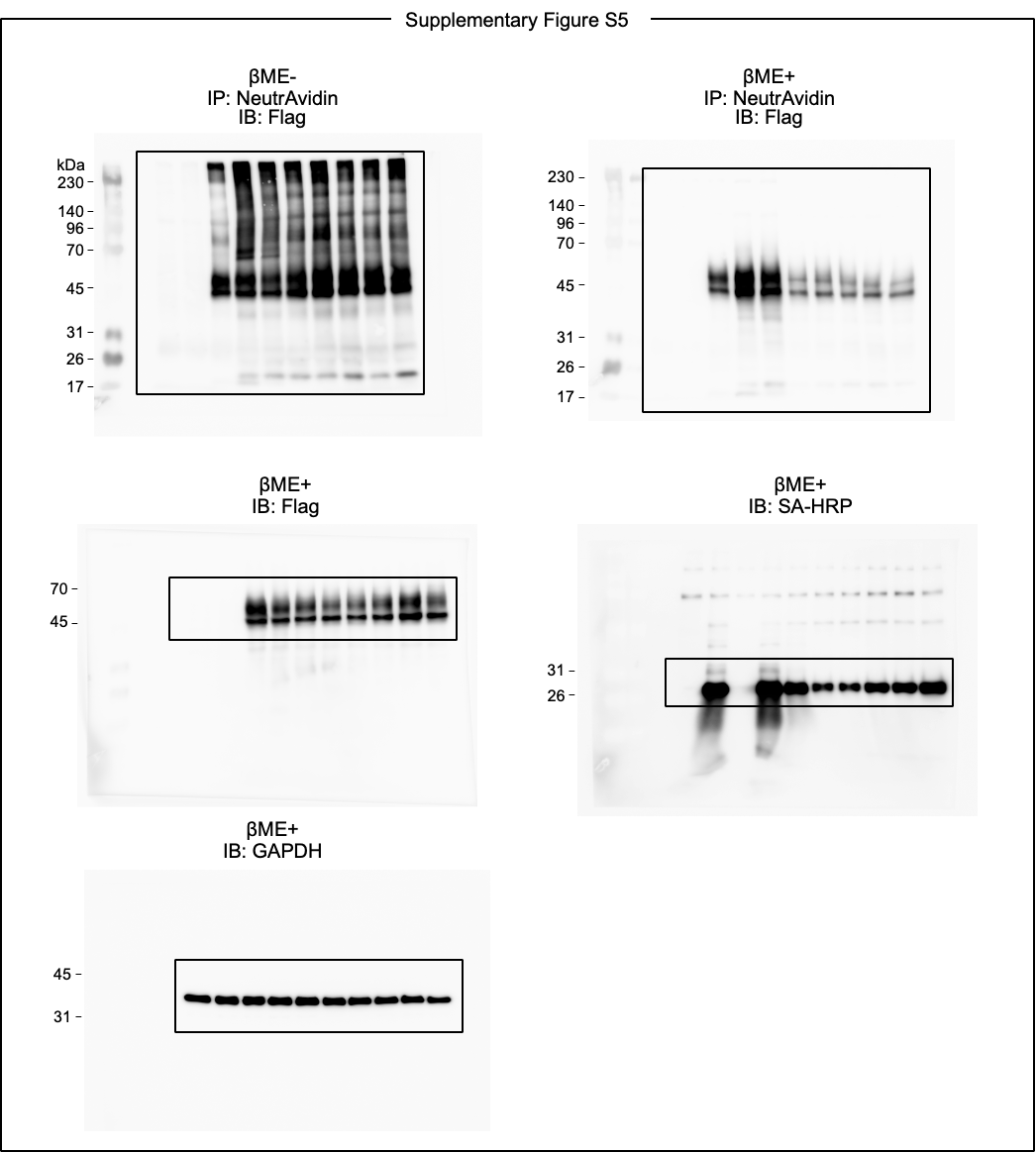
**

**Supplementary Figure S8 Representative entire images of immunoblot.**

Boxed areas were cropped for designated figures.
